# Determining phase coherence time of stored light in warm atomic vapor

**DOI:** 10.1038/s41598-017-15469-4

**Published:** 2017-11-14

**Authors:** Taek Jeong, Jumi Park, Han Seb Moon

**Affiliations:** Department of Physics, Pusan National University, Geumjeong-Gu, Busan, 46241 South Korea

## Abstract

In quantum memory based on an atomic medium, we may have a question about whether all information on the stored light is preserved. In particular, the phase coherence between the stored and retrieval light pulses is very interesting, because it can indicate the relationship between the coherence time and storage time of the light. In this paper, we investigate the phase coherence time of light stored in a warm atomic vapor, by examining the beat-note interference between the retrieval light pulse and a reference light beam optically delayed using an optical fiber. The beat-note interference fringes are measured for different reference-light optical delays. The observed retrieval-light phase indicates that the phase of the input probe light is preserved in the medium. However, we further confirm that the retrieval-light phase coherence depends on the phase coherence of the coupling light used for retrieval in the storage process.

## Introduction

The phenomenon of light storage in an atomic medium is widely regarded as important, having application in various fields, such as atomic physics, quantum optics, quantum computing, and quantum communication^1–10^. The fundamental concept employed in atomic light memory is the superposition of atomic states interacting with coherent light. Techniques for the storage and manipulation of the quantum state of an optical light pulse have been successfully developed to facilitate quantum memory in an atomic medium with atomic coherence. In particular, quantum memory is a key technology for long-distance quantum communication, photonic quantum information networks, and on-demand single photon sources^11–13^. The essential characteristics of light storage are the preservation of various types of information concerning the light, such as its intensity, longitudinal phase coherence, spatial coherence, polarization, and optical frequency^14–17^. Quantum memory is based on maintenance of the quantum properties of the stored light, such as retention of its entanglement, qubits, and photon statistics^17–20^.

From the perspective of classical and quantum optics, the coherence of light is a very important characteristic, because of the associated interference and superposition behavior. Therefore, preservation of the longitudinal phase and transverse spatial coherences of light during a light storage process in an atomic medium has been reported^14–16^. In particular, previous studies have described preservation of the longitudinal phase coherence of a retrieval light pulse in an atomic vapor cell^14^ and a cold atomic medium^15^. Although preservation of the properties of the retrieval light pulse in light storage and quantum memory has been studied^14,15^, the phase coherence of the retrieval light pulse has never been directly compared to that of the input light. Further, the retrieval-light-pulse phase coherence is influenced by the phase coherence of the coupling light beams used for writing and reading in atomic light storage based on electromagnetically induced transparency (EIT); however, this influence has never been considered.

In this paper, we investigate the longitudinal phase coherence of a retrieval light pulse according to the storage and delay times of a reference light in a warm Rb atomic vapor. By comparing the longitudinal phase coherence of the probe light and coupling light with that between the storage and retrieval light pulses using a delayed beat-note interferometer, we directly demonstrate that the longitudinal phase coherence of the retrieval light pulse is dependent on the phase coherence of the coupling light beams used for writing and reading in EIT-based atomic light storage.

## Results

To directly measure the phase coherence of the retrieval light pulse in the atomic light storage, we used a delayed beat interferometer, which yields the beat fringe between the probe light and the delayed reference light. The characteristics of this fringe are influenced by the time delay. The reference light, which was shifted by 2 MHz from the optical frequency of the probe laser, was sent to an optical delay line consisting of three 400-m-long single-mode fibers (SMFs). After passing through the optical delay line, the reference light was combined with the retrieval light pulse at a beam splitter (BS). We measured the beat fringes between the reference light and the retrieval light pulse for several different optical delay lengths.

### Experimental setup

**Figure 1:**
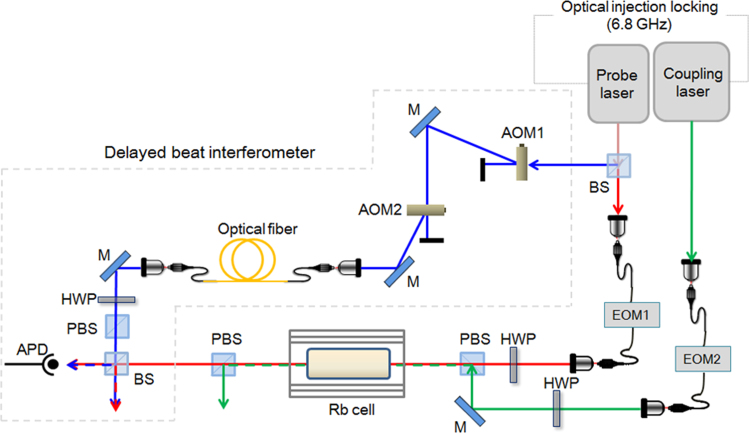
Schematic of experimental apparatus for examining the phase coherence of a retrieval light pulse in light storage featuring warm Rb atomic vapor (AOM: acousto-optic modulator; EOM: electro-optic modulator; PBS: polarization beam splitter; BS: beam splitter, HWP: half-wave plate; M: Mirror; and APD: avalanche photodiode).

A schematic of the experimental apparatus used to examine the phase coherence of the retrieval light pulse is shown in Fig. 1. Two external cavity diode lasers (ECDLs), one for the probe and reference lights and another for the coupling light, were optically locked using an electro-optic modulator (EOM) of 6.8 GHz, which is equal to the hyperfine splitting frequency of the 5S_1/2_ ground state of the ^87^Rb atom. The spectral widths of the ECDLs were estimated to be less than 1 MHz and the relative frequency jittering of the two optically locked lasers was measured to be less than 2 Hz, corresponding to the measurement limitation of the radio-frequency (RF) spectrum analyzer.

The frequencies of the probe and coupling lasers were locked to the 5S_1/2_ (F = 1)–5P_1/2_ (F′ = 1 and 2) transition of ^87^Rb. The probe laser was split into two parts by a BS, yielding the probe and reference light pulses. Thus, one part was the probe light pulse, which was given a half-Gaussian shape using a fiber EOM (EOM1), and the other was the reference light, which was shifted by 2 MHz using two acousto-optic modulators (AOMs) for the beat interferometer. The coupling laser was a control light for storage (writing) and retrieval (reading) in the atomic medium using another fiber EOM (EOM2). The lasers were linearly polarized in the perpendicular direction, and the optical powers of the probe and coupling lasers were 20 μW and 204 μW, respectively. The probe light pulse was combined with the co-propagating coupling light at a polarizing beam splitter (PBS). For the storage medium, we used an Rb vapor cell that was 2.5 cm in diameter and 5 cm in length with a 4-Torr Ne buffer gas. To shield the vapor cell from the Earth’s magnetic field, it was wrapped in three layers of μ-metal sheets. The measured residual magnetic field in the μ-metal chamber was sub-milliGauss. The temperature of the vapor cell was controlled at 45 °C, corresponding to an atomic number density of 1.5 × 10^11^/cm^3^. Following storage in the vapor cell, the retrieval light pulse was measured using an avalanche photodiode (APD).

### Longitudinal phase coherence of retrieval light in atomic medium

**Figure 2:**
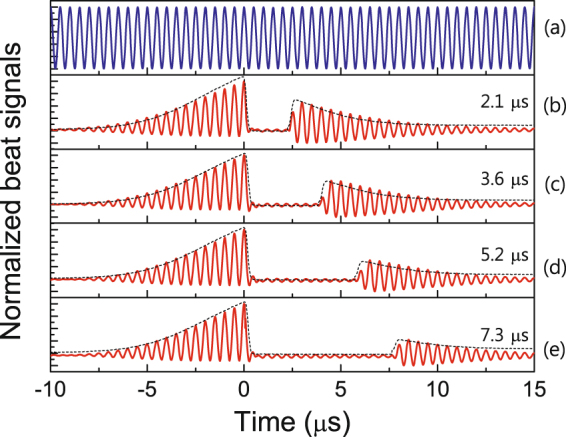
Beat interferometer of the retrieval light in the atomic medium. (**a**) Beat fringe between CW probe light and CW reference light. Beat fringes for storage times of (**b**) 2.1 μs, (**c**) 3.6 μs, (**d**) 5.2 μs, and (**e**) 7.3 μs during the storage and retrieval processes.

To investigate the longitudinal phase coherence of the retrieval light in the atomic medium, we used the beat interferometer between the probe light and reference light. All beat signals in our experiment were averaged over 512 traces. First, we observed the beat fringe between the continuous-wave (CW) probe light and CW reference light, as shown in Fig. 2(a). The visibility *V* of the normalized beat fringe of Fig. 2(a) was estimated to be 1.0 and its frequency was set to correspond exactly to the 2-MHz frequency shift of the reference light achieved using the two AOMs. All beat signals of Fig. 2 were measured when a 10-m-long delay line was used for the reference light. The time delay between the probe light and reference light was estimated to be approximately 50 ns from the optical path length of the delay line. The time delay was considerably shorter than the coherence time of the ECDL used in this setup. However, the coherence time of our ECDL was estimated to be more than 1 μs. As shown in Fig. 2(a), the beat-fringe *V* was high and clear, because the delay time between the two light beams was shorter than the light-source coherence time.

The probe pulse light and the coupling light were operated according to the sequence for storage and retrieval in the atomic medium; hence, the normalized signals of the storage light and retrieval light and the beat fringes of the incoming and outgoing light beams were obtained for various storage times. In our experiment, the probe pulse was shaped to a Gaussian pulse of 5.5 μs at full width at half maximum (FWHM). The coherence time at 27.5% efficiency was estimated to be 7.3 μs. The black dashed curves and red solid curves of Fig. 2(b–e) show the normalized signals of the storage light and retrieval light and the beat fringes of the incoming and outgoing light beams for storage times of 2.1 μs, 3.6 μs, 5.2 μs, and 7.3 μs, respectively. It is apparent that the magnitude of the retrieval light beam decreases when the storage time increases. However, the visibility of the retrieval-light beat signal remains at approximately 1.0 to the retrieval-light magnitude. Further, compared with the beat fringe of the CW probe in Fig. 2(a), the phases of the beat fringes over the entire region of the light storage process are exactly identical to that of the beat fringe of the CW probe light. In particular, in the case of Fig. 2(e), the storage time is longer than the ECDL coherence time, but the retrieval-light beat signal can be observed clearly. Notably, the phase of the retrieval light pulse is consistent with that of Fig. 2(a), which indicates that the retrieval light is coherent with the reference light generated after the storage time. If the implication of this result is accepted, the coherence time of the storage light pulse can be changed and increased by adjusting the storage time. However, it is not clear whether the observed phase of the stored light pulse is determined by preservation of the longitudinal phase coherence of the input light pulse during the storage time, or by its time evolution during storage. Therefore, we must compare the retrieval light pulse with a time-delayed reference light beam, where the reference light beam at a storage moment is first delayed in an optical fiber during the storage time.

### Beat fringes of delayed beat interferometer according to delay-line length

**Figure 3:**
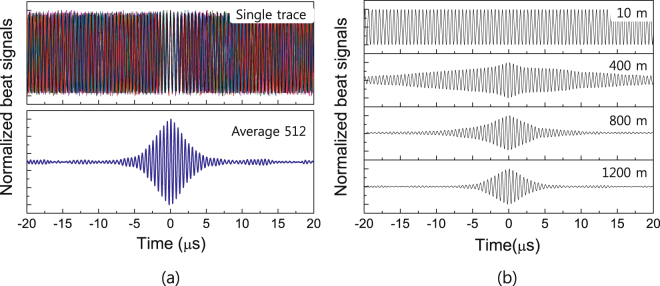
Beat fringes of the delayed beat interferometer according to delay-line length. (**a**) For the time delay of the 1200-m-long delay line, (top) single-shot beat fringes and (bottom) beat signal averaged over 512 traces. (**b**) Delayed beat interferometer fringes according to delay-line length, for delay line consisting of a 10-m and three 400-m-long SMFs, such as 10 m, 400 m, 800 m, and 1200 m.

Before investigating the phase coherence of the retrieval light pulse according to the reference-light time delay, we measured the beat fringes of the delayed beat interferometer. Figure 3 shows the beat fringes between the CW probe light and CW reference light for a 1200-m-long delay line, for which the time delay was estimated to be approximately 6 μs. As shown in the top part of Fig. 3(a), when the beat signals were trigged at a given time, dynamic variation of the beat-fringe phases occurred; this was because of the long time delay between the two light beams compared with the ECDL coherence time. When the beat signals were averaged over 512 traces, a delayed beat fringe was observed, as shown in the bottom part of Fig. 3(a). The magnitude of the delayed beat signal decreased rapidly and vanished within approximately 7 μs.

Figure 3(b) shows the delayed beat interferometer fringes according to the delay line length, for delay lines consisting of combinations of one 10-m-long SMF or up to three 400-m-long SMFs, yielding overall delay line lengths of 10 m, 400 m, 800 m, and 1200 m. The results indicate that, as the delay-line length increases, the maintenance time (*τ*_b_) of the beat fringe decreases; that is, *τ*_b_ is in inverse proportion to the delay-line length. The time delay of each delay line was calculated from the optical path length, corresponding to 0.05 μs, 2.08 μs, 4.15 μs, and 6.23 μs for delay-line lengths of 10 m, 400 m, 800 m, and 1200 m, respectively. The corresponding FWHM values of the delayed beat fringes were estimated to be 25 ms, 20 μs, 6.0 μs, and 4.4 μs, respectively. Thus, we next investigated the phase coherence of the incoming light and outgoing light during the storage time.

### Phase coherence of retrieval light pulse according to reference-light time delay

**Figure 4:**
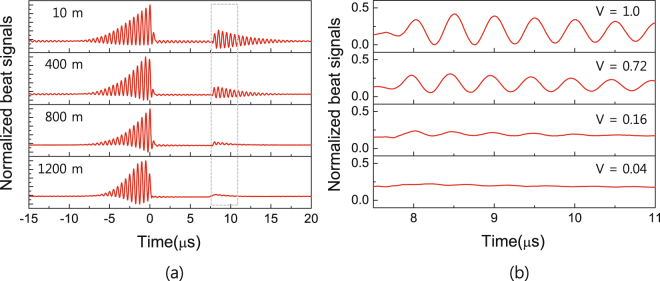
Beat fringes of the retrieval light pulse according to the delay-line lengths of the reference-light. (**a**) Phase dynamics of storage and retrieval lights according to reference-light time delay during storage process with 7.3-μs storage time. (**b**) Magnified beat signal in portion of retrieval light pulse of Fig. 4(a).

To examine the preservation of the phase coherence of the input light pulse during the storage time, we compared the retrieval-light phase with that of the reference light for various time delays. The time delay between the retrieval light and reference light can be controlled by changing the delay-line length. Figure 4(a) shows the phase dynamics of the storage and retrieval light beams according to the reference-light time delay during the storage process, for a 7.3-μs storage time. Interestingly, Fig. 4(a) indicates that the retrieval-light beat fringe decreases and averages out as the delay-line length increases.

To clearly confirm the beat fringe removal for the retrieval light, we magnified a portion of the retrieval light pulse of Fig. 4(a), as shown in Fig. 4(b). When the visibility *V* of the beat fringe for the 10-m-long delay line was set to a reference value of *V* = 1, the *V* values of the delayed beat fringes were estimated to be 0.72, 0.16, and 0.04 for delay-line lengths of 400, 800, and 1200 m, respectively. The results of Fig. 4 mean that the retrieval-light phase is not identical to that of the incoming probe light. Therefore, it seems that the phase coherence of the input probe light is not preserved during the storage time.

However, let us consider the phase of the coupling light used for writing and reading in the atomic medium. Basically, EIT-based atomic light storage utilizes the superposition of atomic states interacting with the probe light and coupling light. When the probe light is used for writing to the atomic medium, atomic coherence is generated by both the probe light and coupling light. Information on not only the probe light, but also the coupling light is stored in the atomic medium. When the information of the stored probe light is read from the atomic memory, it is read from the atomic-medium memory by the coupling light only. However, the coupling-light phase when the probe light pulse is being used to write to the atomic medium differs from that during reading from the atomic medium. Therefore, the phase coherence of the coupling light for reading from the atomic light storage affects the retrieval-light phase.

The experimental results of Fig. 4 are understood as indicating the phase coherence between writing and reading for the coupling light beams. First, in the case of the 10-m-long delay line, it was found that the coupling light generated after the 7.3-μs storage time read the stored probe light from the atomic medium; however, a high-*V* beat signal appeared despite the over-limitation of the coherence time of the employed laser. This beat fringe was due to interference between the retrieval light and the reference light generated after the 7.3-μs storage time. Note that, if the retrieval-light phase coherence corresponds to that of the writing coupling light, the mechanism behind the maintenance of the high *V* and continuity of the phase of the retrieval-light beat fringe, which is independent of the storage time, can be understood. That is, this behavior occurs because the time delay between the retrieval light and reference light is considerably shorter than the light-source coherence time.

Second, in the case of the 1200-m-long delay line, when the retrieval-light phase was compared with that of the input probe light, the retrieval-light beat signal was averaged out. The low *V* of the beat fringe between the retrieval light and reference light at a storage moment means that the phases of the incoming light and outgoing light are uncorrelated. This result can be illustrated as the phase correlation between the retrieval and writing coupling light beams, because the time delay between the retrieval and reference light beams is similar to the storage time (6.3 and 7.3 μs, respectively) and considerably longer than the light-source coherence time. Therefore, based on the experimental results of Fig. 4, we have confirmed that the retrieval-light phase coherence is due to preservation of the phase coherence of the input light pulse, while also corresponding to the phase of the coupling light beam used for reading^21,22^.

### Phase coherence of retrieval light pulse according to storage time

**Figure 5:**
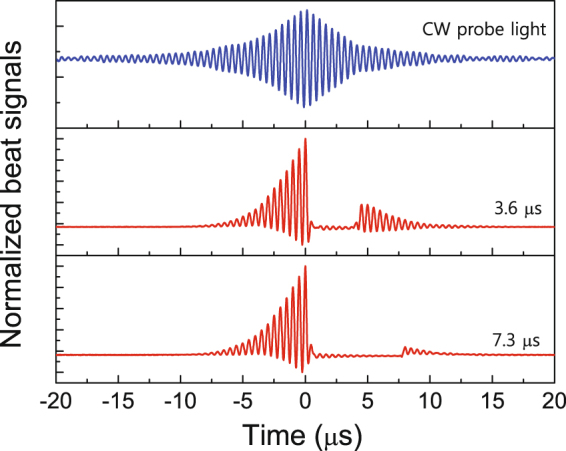
For 800-m-long delay line corresponding to 4.2-μs time delay, beat fringes of CW probe light and storage light pulses according to storage time.

For a reference-light delay line with a length fixed at 800 m, corresponding to a time delay of 4.2 μs, we investigated the retrieval-light phase for storage times of 3.6 μs and 7.3 μs. Figure 5 shows the beat fringes of the CW probe light and storage light pulses, allowing analysis of the retrieval-light phase coherence compared with the beat fringe of the CW probe light. When the storage time was increased from 3.6 μs to 7.3 μs, the magnitude and visibility of the retrieval-light beat signal decreased, as shown in Fig. 5. Comparing the beat fringes of the CW probe light and storage light pulses, it is apparent that the beat signal visibility of the retrieval light is related to the line shape of the beat-fringe envelope of the CW probe light. The amplitude of the beat fringe of the CW probe light affects the beat signal visibility of the retrieval light at a given storage time. However, the beat-fringe phase of the light storage process corresponds to that of the CW probe light. When the time delay between the writing coupling light and reference light is determined, the properties of the beat fringe between the retrieval light and reference light correspond to those of the beat fringe of the CW probe light, because the longitudinal phase coherence of the retrieval light pulse is dependent on that of the reading coupling light^21^.

**Figure 6:**
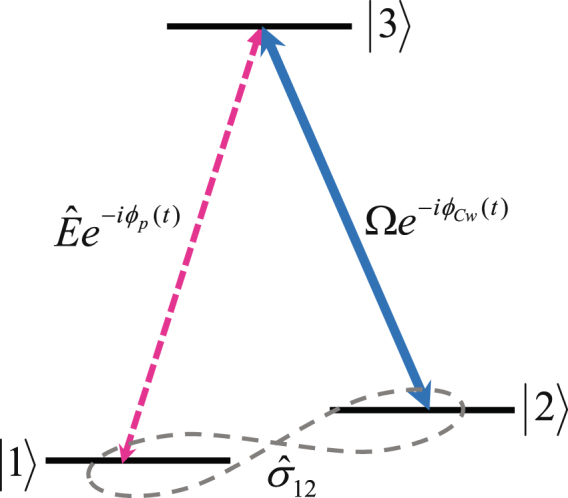
Λ-type three-level atomic model with the probe field $\hat{E}(z,t){e}^{-i{\phi }_{p}(t)}$ and coupling field ${\rm{\Omega }}{e}^{-i{\phi }_{Cw}(t)}$ for writing process; the generated atomic coherence ${\hat{\sigma }}_{12}$ between two ground states.

In order to further understand our results that the longitudinal phase coherence of the retrieval light depend on that of the reading coupling light, we briefly illuminated as the dark-state polariton (DSP) in a Λ-type atomic system^23^, whose schematic diagram is shown in Fig. 6. The three-level atomic model in Fig. 6 is composed of two ground states ($|1\rangle$ and $|2\rangle$) and an excited state ($|3\rangle$). The density matrix equation of motion can be expressed as

1 $$\frac{\partial {\rho }_{ij}}{\partial t}=-\frac{i}{\hslash }\sum _{k}[{H}_{ik}{\rho }_{kj}-{\rho }_{ik}{H}_{kj}]-{{\rm{\Gamma }}}_{ij}{\rho }_{ij},$$

where the subscript indices *i* and *j* indicate the $|i\rangle$ and $|j\rangle$ states, respectively. *ρ*_*ij*_ denotes a density-matrix element and *H*_*ij*_ the effective interaction Hamiltonian, which is composed of the atomic and interaction Hamiltonians. Further, the decay rate between the $|i\rangle$ and $|j\rangle$ states is represented by Γ_ij_. ρ_12_ is a term of two-photon coherence between two ground states in Λ-type configuration. It is convenient to transform into a co-rotating frame to eliminate the fast rotation. We can transform the density-matrix elements (*ρ*_*ij*_) into the rotating frame of a slowly varying density operator (${\hat{\sigma }}_{ij}$). The atom-light interaction Hamiltonian in rotating frame can be written as

2 $${\hat{H}}_{IN}=-\frac{N}{L}\int dz[\hslash g{\hat{E}}^{+}(z,t){\hat{\sigma }}_{13}(z)+\hslash {{\rm{\Omega }}}^{\ast }(t){\hat{\sigma }}_{23}(z)+h.c.],$$

where *N* and *L* represent the number of interaction atom and the length of atomic ensemble in the z-direction (propagation direction of the probe field), respectively. *g* denotes the coupling constant between the $|1\rangle$ and $|3\rangle$ states. $\hat{E}(z,t)$ is written as probe field operator coupled to the transition between the $|1\rangle$ and $|3\rangle$ states and ${\rm{\Omega }}(t)$ is the Rabi frequency of the coupling field coupled to the transition between the $|2\rangle$ and $|3\rangle$ states.

However, to consider the influence on the retrieval-light-pulse phase coherence of the phase coherence of the coupling light, we distinguished the phases of the coupling fields for writing and reading in atomic light storage. ${\varphi }_{p}(t)$ and ${\varphi }_{Cw}(t)$ denote the phase fluctuations of the initial probe field and the coupling fields for writing. Under weak field condition, the steady-state two-photon coherence ${\hat{\sigma }}_{12}$ in the adiabatic limit can be written as

3 $${\hat{\sigma }}_{12}(z,t)=-g\frac{\hat{E}(z,t)}{{\rm{\Omega }}(t)}{e}^{-i[{\varphi }_{p}(t)-{\varphi }_{Cw}(t)]}.$$

As is well-known, the probe field is contributed to generate the two-photon coherence ${\hat{\sigma }}_{12}$ with the coupling field. Under our experimental conditions of the phase-locked probe and coupling fields for writing process, the relative phase ${\phi }_{p}(t)-{\phi }_{Cw}(t)$ of ${\hat{\sigma }}_{12}$ is zero. From the propagation equation of the quantum field in the storage and retrieval process, the DSP $\hat{{\rm{\Psi }}}(z,t)$ during the storage and retrieval process can be expressed as

4 $$\hat{{\rm{\Psi }}}(z,t)=\frac{{g}^{2}N}{\sqrt{{\rm{\Omega }}(t){{\rm{\Omega }}}^{\ast }(t)+{g}^{2}N}}\frac{\hat{E}(z,t)}{{\rm{\Omega }}(t)}{e}^{-i[{\varphi }_{p}(t)-{\varphi }_{Cw}(t)]}+\frac{{\rm{\Omega }}(t)}{\sqrt{{\rm{\Omega }}(t){{\rm{\Omega }}}^{\ast }(t)+{g}^{2}N}}\hat{E}(z,t){e}^{-i{\varphi }_{Cr}(t)},$$

where ${\phi }_{Cr}(t)$ denotes the phase fluctuations of the coupling fields for reading. The first term of Eq. (4) described the matter component for the storage process in the atomic ensemble. The spin state of matter component is independent on the phase fluctuation between probe and coupling fields because of the phase-locked probe and coupling fields. However, when the coupling field for reading is turn on, as can be known from the second term of Eq. (4), the phase of the retrieval field is identical to that of the coupling light for reading. Therefore, we can conclude that the retrieval-light phase coherence corresponds to the phase of the coupling light for reading.

## Conclusion

We have reported the dependence of the longitudinal phase coherence of a retrieval light pulse on the phase of the coupling light used for reading during the retrieval process, for the case of a warm Rb atomic vapor employed as the atomic medium. A delayed beat interferometer between the retrieval light and a delayed reference light was employed to analyze the retrieval-light phase. From the visibility and phase of the observed beat fringe between the retrieval and delayed reference light beams for various delay times, we experimentally confirmed that the phase coherence of the retrieval light to the delayed reference light is dependent on the phase of the coupling light used for reading in the storage process. Our experimental results demonstrate that the retrieval-light phase coherence is preserved for the storage time, but corresponds to that of the coupling light for reading. As the properties of the retrieval light are related to not only the incoming probe light, but also the coupling light, our results have wide application with regard to the classical and non-classical properties of retrieval light, such as its spatial coherence, polarization, optical frequency, entanglement, qubits, and photon statistics. We further believe that our results have very important implications for various fields related to light storage and quantum memory, such as atomic physics, quantum optics, and quantum communications.

## References

[1] Liu C, Dutton Z, Behroozi CH, Hau LV (2001). Observation of coherent optical information storage in an atomic medium using halted light pulses. Nature.

[2] Gorshkov AV (2007). Universal approach to optimal photon storage in atomic media. Phys. Rev. Lett..

[3] Novikova I (2007). Optimal control of light pulse storage and retrieval. Phys. Rev. Lett..

[4] Appel J, Figueroa E, Korystov D, Lobino M, Lvovsky AI (2008). Quantum memory for squeezed light. Phys. Rev. Lett..

[5] Choi KS, Deng H, Laurat J, Kimble HJ (2008). Mapping photonic entanglement into and out of a quantum memory. Nature.

[6] Lvovsky AI, Sanders BC, Tittel W (2009). Optical quantum memory. Nat. Phot..

[7] Jensen K (2011). Quantum memory for entangled continuous-variable states. Nat. Phys..

[8] Specht HP (2011). A single-atom quantum memory. Nature.

[9] Chen Y-H (2013). Coherent optical memory with high storage efficiency and large fractional delay. Phys. Rev. Lett..

[10] Hosseini M, Campbell G, Sparkes BM, Lam PK, Buchler BC (2011). Unconditional room-temperature quantum memory. Nat. Phys..

[11] Duan L-M, Lukin MD, Cirac JI, Zoller P (2001). Long-distance quantum communication with atomic ensembles and linear optics. Nature.

[12] Kimble HJ (2008). The quantum internet. Nature.

[13] Chen S (2006). Deterministic and storable single-photon source based on a quantum memory. Phys. Rev. Lett..

[14] Mair A, Hager J, Phillips DF, Walsworth RL, Lukin MD (2002). Phase coherence and control of stored photonic information. Phys. Rev. A.

[15] Chen Y-F, Liu Y-C, Tsai Z-H, Wang S-H, Yu IA (2005). Beat-note interferometer for direct phase measurement of photonic information. Phys. Rev. A.

[16] Lee J-C, Park K-K, Cho Y-W, Kim Y-H (2013). Preservation of spatial coherence of an optical pulse in atomic vapor quantum memory. Phys. Rev. A.

[17] Cho Y-W, Kim Y-H (2010). Atomic vapor quantum memory for a photonic polarization qubit. Opt. Express.

[18] Chanelière T (2005). Storage and retrieval of single photons transmitted between remote quantum memories. Nature.

[19] Zhang H (2011). Preparation and storage of frequency-uncorrelated entangled photons from cavity-enhanced spontaneous parametric downconversion. Nat. Photonics.

[20] Bae I-H, Moon HS (2012). Transfer of photon number statistics from coupling light to stored and retrieved probe light. Opt. Express.

[21] Park K-K, Zhao T-M, Lee J-C, Chough Y-T, Kim Y-H (2016). Coherent and dynamic beam splitting based on light storage in cold atoms. Sci. Rep..

[22] Jeong T, Moon HS (2016). Phase correlation between four-wave mixing and optical fields in double Λ-type atomic system. Opt. Express.

[23] Fleischhauer M, Lukin MD (2000). Dark-state polaritons in electromagnetically induced transparency. Phys. Rev. Lett..

